# The central nervous system in childhood chronic kidney disease

**DOI:** 10.1007/s00467-006-0269-1

**Published:** 2007-10-01

**Authors:** Debbie S. Gipson, Peter J. Duquette, Phil F. Icard, Stephen R. Hooper

**Affiliations:** 1 UNC Kidney Center, University of North Carolina School of Medicine, 7012 Burnett Womack Bldg., CB# 7155, Chapel Hill, NC 27599-7155 USA; 2 School of Education, University of North Carolina, Chapel Hill, NC USA; 3 Clinical Center for Development and Learning, University of North Carolina School of Medicine, Chapel Hill, NC USA; 4 Department of Psychiatry, University of North Carolina School of Medicine, Chapel Hill, NC USA

**Keywords:** Kidney failure, Central nervous system, Learning, Education, Children

## Abstract

Neurodevelopmental deficits in pediatric and adult survivors of childhood onset chronic kidney disease (CKD) have been documented for many years. This paper reviews the available literature on central nervous system involvement incurred in childhood CKD. The studies reviewed include recent work in neuroimaging, electrophysiology, and neuropsychology, along with commentary on school functioning and long-term outcomes. The paper concludes with suggestions for monitoring the neurodevelopmental status and pursuing appropriate early interventions for children with CKD.

The excellent long-term survival for children with chronic kidney disease (CKD) is a strong motivator for optimizing neurodevelopment and educational achievement [1]. Over the course of the past 30 years, reports of advances in CKD therapies have decreased both morbidity and mortality risks. Indeed the identification and elimination of aluminum neurotoxicity and the availability of erythropoietin therapy has eliminated two significant contributors of nervous system dysfunction in patients with kidney failure. Despite this success, the neurodevelopment of children with CKD continues to be impeded by the presence of kidney disease [2]. This review describes the current understanding of the nervous system structure and function in children with CKD and highlight areas where additional study is needed.

## Neuroimaging

Investigations into neurodevelopmental outcomes in CKD pediatric cohorts were sparked by seminal brain imaging studies of the early 1980s, which found several cortical abnormalities in this population. Researchers utilized computed tomography (CT) scans and magnetic resonance imaging (MRI) to verify the neuroanatomical abnormalities such as cerebral atrophy and infarcts in children with ESRD [3–6]. Cerebral atrophy has been documented in 12–23% of children with ESRD [4, 5, 7]. Research has focused on certain disease groups at greater risk for CNS dysfunction than the general CKD population, including cohorts of congential nephrotic syndrome, cystinosis and Lowe syndrome. Valanne et al. [7] reported the MRI findings of 33 patients, comprised mostly of children with congenital nephrotic syndrome between 6 and 11 years of age, who received a kidney transplant prior to 5 years of age [7]. Results indicated that 18 of 33 (54%) patients had chronic infarct lesions mostly within the periventricular white matter. Comparable to previous findings, cerebral atrophy was found in five of 33 (15%) patients [5, 7]. Evidence of white matter lesions in this sample of patients with congenital nephrotic syndrome was highly correlated with transplantation at a later age (*P*<0.05), longer duration of dialysis (*P*<0.02), and a history of hemodynamic crisis (*P*<0.03) [7]. Findings by Nichols et al. [8] on pediatric patients with cystinosis from infancy showed that ten of 11 (91%) patients had cortical atrophy. Case studies on CNS and renal involvement of patients with Lowe syndrome have also suggested the presence of cerebral atrophy and seizure disorders [9]. In summary, children with CKD are at risk for central nervous system (CNS) structural abnormalities of atrophy and infarcts. These lesions may be observed with greater frequency in populations at risk for coagulation disorders such as congenital nephrotic syndrome and among those with a history of hypertensive crisis. The overall risk for CNS structural abnormalities can only be estimated based on currently published data for children with ESRD. Additional research into the risks for CNS structural abnormalities in children with less severe CKD and a broader scope of the general CKD population will be necessary for advances in this field.

## Electrophysiology

In addition to structural abnormalities, children with CKD have also shown deficits in cerebral and peripheral nerve conduction. Measurement techniques in this area include electroencephalography (EEG) measuring cortical electrical activity; brain stem evoked responses measuring brainstem response to stimuli in both timing and intensity; and peripheral nerve conduction assessing peripheral nerve function. Four reports of EEG findings have been published since 1990 in the pediatric CKD literature. In a report on patients with CKD from infancy, results identified unspecified EEG abnormalities in six of 14 (42%) patients but no abnormalities were found with brainstem auditory evoked potentials [4]. Similar rates of EEG abnormalities (12 of 33, 36%) have been reported for patients after renal transplant, all of whom had ischemic watershed zone lesions on MRI images of the CNS [5]. Hurkx et al. [10] also evaluated 22 children with CKD. Brainstem conduction in the auditory pathway was within normal limits and did not differ between the peritoneal dialysis and chronic renal insufficiency (CRI) subgroups. However, results did suggest the possibility of delayed myelination or synaptogenesis of the somatosensory pathway in young children with CKD regardless of renal replacement therapy [10]. This finding was explained by deficits in cortical conduction via the thalamus as measured by high interpeak somatosensory evoked potential latencies, which suggests that earlier onset of CKD pathology could hinder maturation of the myelin sheath in the somatosensory cortex. Contemporary estimates of the frequency of seizure disorders among children with CKD are not known.

**Fig. 1:**
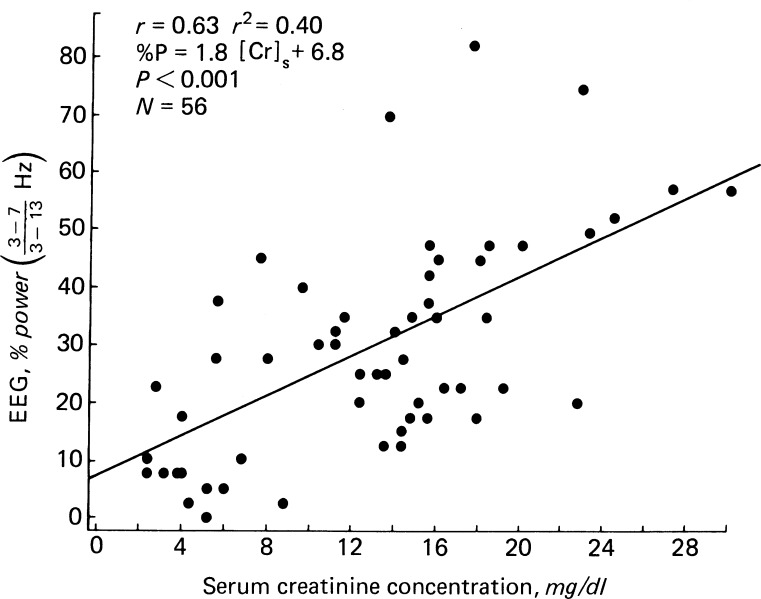
Plot of relationship between EEG slowing and serum creatinine. EEG frequency is defined as the number of slow waves (3–7 Hz) divided by the total waves (3–13 Hz), expressed as percent. (Reprinted from [11] with kind permission from Macmillan Publishers)

Research on adults with CKD expands our understanding of the relationship between severity of kidney disease and electrophysiology. A progressive slowing of EEG waves is correlated with progressive elevation in serum creatinine [11] (see Fig. 1). Additional evaluation of the relationship between evoked potential and the severity of anemia of CKD confirms a slow and diminished intensity of CNS response to stimuli with anemia. This CNS function improves with correction of anemia with erythropoietin therapy in adults with CKD. Similar data in pediatric patients are not available [11–15].

Assessment of peripheral nerve conduction function in children is supported by long-standing documentation of abnormalities in this area in adults with CKD. A single study has measured peripheral nerve conduction using the median nerve in ten children on hemodialysis between the ages of 9 and 19 years. Comparing their performance to age- and gender-matched controls, abnormal peripheral conduction velocities were documented in three of ten (30%) children [16].

### Summary

The published reports of children with CKD have suggested non-specific EEG abnormalities, delayed myelination of the somatosensory cortex and slower peripheral nerve conduction velocities. The frequency, severity and risk factors for these abnormalities among children with mild to severe CKD are unknown and will require research including a diverse cohort. The extreme of EEG abnormalities may be expressed as seizure disorders or encephalopathy. Contemporary estimates of the frequency of these abnormalities will also require additional investigation.

## Neurodevelopmental outcomes in children with CKD

In addition to the assessment of CNS structure and nerve conduction, the functional status of the developing child is assessed though neuropsychological testing and educational achievement. Cross-sectional studies of children with CKD suggested that there is increased risk for delays in neurocognitive development, particularly for toddlers [17–19] and children with end-stage renal disease (ESRD) [17–23]. Unfortunately, no large-scale, prospective neurocognitive evaluation of this health-challenged group has been done to allow for an accurate prediction of the incidence, prevalence, and magnitude of developmental problems. The studies to date have used modestly sized cohorts to examine general neurocognitive functions as well as more specific neurocognitive domains, including attention, executive function, language, visual-spatial abilities and memory (see Table 1).

**Table 1:** Summary of testing used for developmental assessment in children

Neurocognitive function	Definition
IQ	A measure of an individual’s overall or general cognitive functioning. IQ scores are typically used for school-age children to assess both verbal and non-verbal reasoning abilities. Developmental screeners are typically used for children under 6 years. Developmental screeners are used to assess motor skills, language, and visual reception.
Attention	A multi-component construct that requires selectively attending to and sustaining focus on a stimuli for a period of time, while blocking out distractors. Attention can be assessed for both visual and auditory stimuli.
Executive function	Problem-solving process consisting of several components: (1) planning, (2) initiating, (3) goal formulation, (4) set-shifting, and (5) behavioral inhibition. Executive functions are highly correlated with attention.
Language	Ability to communicate verbally (Expressive Language) and to understand spoken language (Receptive Language).
Visual-spatial ability	The ability to accurately perceive visual information in two- and three-dimensional form. Visual-spatial tasks require localization, orientation, integration, and construction of visual information.
Memory	The process of encoding, processing, storing, and retrieving visual or verbal stimuli. Information may be stored for short (Short-Term Memory) or long periods of time (Long-Term) memory. Working memory requires and individual to perform a mental operation on information stored in short-term memory such as repeating a series of verbally-presented numbers backwards.
Academic achievement	Performance across traditional academic domains, including basic reading, reading comprehension, math reasoning, math calculation, and written language.

### General neurocognitive function

Developmental quotients (DQ) and intelligence quotients (IQ) are used as markers of general developmental status in preschool and school-age children, respectively. In children younger than 5 years of age, neurodevelopmental testing has revealed a number of concerns pertaining to initial developmental trajectories. Hulstijn-Dirkmaat et al. [24] compared the general development of 15 toddlers with chronic renal insufficiency (CRI) receiving conservative therapy with 16 dialysis-dependent children. These investigators found that the children with CRI had a significantly better developmental index as a group when compared with the dialysis-dependent children, with the dialysis-dependent children manifesting a mean developmental index that fell within the mild range of mental retardation. Ledermann et al. [25] evaluated the long-term outcome of infants requiring peritoneal dialysis, with about 25% of the sample evidencing developmental delay. Warady et al. [26] evaluated 28 dialysis dependent infants at 1 year of age, with about 21% being in the low-average to impaired range of general development. Taken together, these studies suggest that approximately 20–25% of very young children with ESRD show general developmental delays and neurodevelopmental impairment is greater with a greater severity of kidney disease.

For older children with CKD, more formal measures of intelligence, IQ, can be obtained. In general, school-age children with CKD show an IQ distribution that is shifted slightly downward compared with the normal population [5, 22, 24, 27, 28]. The children with ESRD tend to have the lowest IQ when compared with siblings or a healthy control group in total IQ and verbal and performance IQ subscales [23, 29]. When comparing IQ within groups of children with CKD, variable results have been reported. Lawry et al. [28] evaluated 13 transplanted children compared with 11 dialysis-dependent children in a cross-sectional study and found a higher mean IQ in the transplant group, although both groups fell largely within the average range. Conversely, a cross-sectional study by Brouhard et al. [23] found no significant differences in the intellectual functioning of their transplant versus dialysis-dependent groups. Warady et al. [26] reported IQ within the normal range for 79% of his sample of 6.6 year-old transplant recipients who had ESRD from infancy. Warady and co-workers also noted that 72% achieved average verbal IQ, while only 56% had an average nonverbal IQ.

#### Summary

It appears that general cognitive functioning scores are lower than normal among children with CKD. The available literature is inadequate to fully characterize this effect with respect to timing of onset of CKD, degree of kidney dysfunction, or differences between verbal and nonverbal abilities and further research is needed in this area.

### Specific neurocognitive functions

#### Attention and executive functions

The ability to attend to a task or classroom environment includes the focus on the task at hand and the suppression of distracting noises and events. Attention has been compared in children with severe CKD before and after transplant [22]. Improvements in sustained attention and mental processing speed 1 year after transplant was observed in the small sample of nine children, comparing before and after transplant functioning. Further, Qvist et al. [5] reported no group deficits of attention in a renal transplant sample when compared with the normative population; however, 24% of their sample showed generalized attention deficits.

Although highly correlated with attention, executive function measures higher order brain function that includes problem solving. Using a multidimensional model of executive functioning (i.e., Initiating, Sustaining, Set-Shifting, Inhibiting), Gipson et al. [30] recently showed the presence of significant problems in selected executive functions in their sample of children with CKD when compared with a typically developing comparison group. The CKD group did not include transplant recipients. The CKD group performed poorer than the typical group in the Initiation and Sustaining domains, controlling for chronologic age and IQ. The groups did not differ on Set-Shifting, changing from one task to another, or Inhibition. The present literature suggests that children with CKD have deficits in attention in as many as 24% of transplant recipients. The effects on children with severe CRI and dialysis dependence appear greater compared with transplant recipients. Furthermore, selected disruption in beginning and sustaining a task, both of which are considered key components of attention, has been identified.

#### Language

Among children with CKD, the prevalence of hearing loss is approximately 18%, and concerns are raised that unrecognized or delayed diagnosis of hearing impairment may impede language development [5, 31]. This finding also raises additional concerns for the literature in that children with known hearing loss may be excluded from studies of cognitive function, thus the language functioning of children with CKD may not be fully represented in published documents [27]. Fennell et al. [32] found deficits in the verbal abstraction abilities of their children with CKD when compared with a matched control group. In contrast, Qvist et al. [5] documented no deficits in language in their transplanted group when compared with the normative population, with only 6% of children having problems on language tasks. More research is needed within this cognitive domain to determine if language abilities are an area of concern for children with CKD, particularly with respect to potential delays secondary to hearing impairment. Furthermore, the assessment of auditory acuity should be a mandatory component of pediatric CKD management to avert potential language delays that may accompany hearing deficits.

#### Visual-spatial abilities

The comprehension and reproduction of two- and three-dimensional objects is a fundamental cognitive function. Fennell et al. [32] documented deficits in visual-motor abilities in a cohort of 56 children with CKD when compared with a matched typical sample. Similarly, Bawden et al. [29] showed that their group of children with ESRD evidenced significantly lower visuoconstructive abilities when compared with sibling controls. In contrast, Qvist et al. [5] documented no overall group deficits in the visuospatial abilities of their sample of transplant recipients when compared with the normative population; however, nearly one-quarter of their sample did show visuospatial deficits. The current literature supports a concern that visual-spatial abilities are diminished in children with CKD with a greater frequency than what is expected in the normative population.

#### Memory

Although one study has identified no differences in short-term memory comparing children with ESRD and sibling controls [29], the majority of literature suggests that children with CKD have memory deficits. In a heterogeneous sample of children with CKD, Fennell et al. [32] documented lower memory abilities for children with CKD when compared with controls, with these abilities deteriorating over a 1-year time span regardless of treatment modality. Mendley et al. [22] documented improvements in working memory in nine children after transplant when compared with their pre-transplant findings. Qvist et al. [5] reported that 20% of the transplant recipients displayed generalized memory deficits. More recently, using a non-transplant sample of children with CKD, Gipson et al. [30] showed significantly lower memory abilities when compared to healthy children. Specifically, the CKD group demonstrated poorer short-term visual memory, short-term verbal memory, and new learning, with specific concerns being raised for the integrity of working memory functions.

#### Summary

Across these specific domains of cognition, the findings clearly point to significant concerns for the neuropsychological integrity of children with CKD-even following kidney transplant. While several of these domains require more scientific inquiry (e.g., language, visual-spatial), the findings for attention, executive function and memory appear to point to areas for concern. More generally, the contemporary findings to date across both the intellectual and neurocognitive domains would suggest the need for developmental surveillance by the pediatric nephrologist and developmental specialists beginning before the onset of ESRD. How these neuropsychological deficits may impact on life course, educational status, and vocational choices remains to be determined, particularly as such factors may impact upon socioeconomic status [33].

## Academic achievement and school functioning

There are relatively few studies examining the academic achievement and school functioning of children with CKD. When reviewing the cognitive functions summarized above, one might expect limitations in math and reading, based on difficulties with executive functioning and memory domains. In general, the available studies suggest that transplant recipients outperform dialysis-dependent patients in the broad academic areas of reading, math, and language [30]. Brouhard et al. [23] reported findings showing the combined ESRD group (transplant and dialysis) had lower achievement scores than their sibling controls in spelling, math, and reading. In contrast, Bawden et al. [29] demonstrated that children with ESRD showed no group differences on various measures of basic reading, spelling, math, reading comprehension, and phonological processing when compared with their sibling controls.

The educational status of children with CKD will typically reflect the cognitive abilities and adaptive functioning of the student. Just as one can find means to perform at peer level with a physical disability, the adaptive capabilities of a child may allow school functioning beyond the predicted level based on neuropsychologic testing. In North America and Europe, 79–94% of children with CKD receive their education in regular educational settings, while 13–15% received special education services not related to visual or hearing impairments [5, 26]. Findings from the cohort by Qvist and co-workers suggested that ten of 13 (77%) patients receiving special education services had CNS infarcts [5]. The rates of special education service provision for children with CKD are equivalent to national rates among the general population [34]. Given the evidence for neuropsychological impairment across the CKD spectrum, however, these rates may represent an underestimate of the number of children with CKD who may actually require special education services to perform optimally in the school setting [18]. Further research should address this need, while routine clinical care should include a thorough psychoeducational screening and ongoing monitoring of school performance in this population.

## Long-term outcomes

Educational attainment and employment have been examined in follow up investigations of childhood ESRD cohorts. Neuropsychological impairment beginning in children with CKD has been shown to persist into adulthood [29, 35]. A Dutch study found adult survivors of childhood onset ESRD have Verbal IQ, Performance IQ, and Full Scale IQ to be 9.7, 9.2, and 10.4 points (*P* values <0.0001) lower than controls, respectively [35]. In addition, the educational attainment is also much lower for adults with childhood-onset ESRD compared with national population statistics [35]. A retrospective study by Broyer et al. [36] found that only 31.2% of their transplant cohort had received a high school diploma, and 68.8% had a junior high education or less. With the burden of diminished cognitive function and lower levels of education, 50–75% of adults with a functioning transplant and 25–60% of patients on dialysis are employed as adults [37]. Lower rates of employment and low occupational level are associated with dialysis rates longer than 8 years [38]. These rates vary by country, with some countries (e.g., France) showing employment rates similar to the general population [36].

## Assessment and management

A practical approach to the assessment of CNS structure and function may include CNS imaging when indicated by a history of poor cranial growth, hemodynamic instability or prolonged dialysis dependence. EEGs are indicated with a concern for seizure disorders but are not recommended for all patients. Early assessment of cognitive function for all children with severe CKD and ESRD is recommended. This assessment should include a general assessment of cognition using DQ or IQ depending on patient age. Additional testing for specific abnormalities in the domains of memory, attention, executive function and visual-spatial skills will complete the assessment for school-age children. After transplantation, a repeat evaluation may prove useful to define remaining deficits and to restructure specific educational plans. Furthermore, developmental intervention for preschool children and educational support through individual educational plans, tutoring or special classes will likely optimize the developmental and educational outcome for children with CKD.

## Conclusion

Across four decades, medical literature has documented neurodevelopmental deficits in children and adult survivors of childhood onset CKD. The vulnerability of the CNS to atrophy, delayed conduction velocity on electrophysiology studies, and specific cognitive deficits has been identified. Although significant work remains, the current literature provides a rationale for neurodevelopmental monitoring and early intervention for children with CKD. The results of this monitoring should be used to provide appropriate developmental and educational intervention. The association between duration of dialysis and CNS infarcts and cognitive dysfunction should at least be considered when assessing the timing of transplantation for children with ESRD.

## CME questions

### (Answers appear following the reference list)

1. You have been following the medical progress of an 8-year-old male child since his diagnosis of kidney disease at the age of 2 years. Over the past 6 years, the kidney disease has been worsening slowly, but the child has responded nicely to treatment. No developmental problems have been described, although the height of the child is beginning to drop below the 10th percentile for his chronological age and gender. On the current medical visit the child reports no problems with school or with his social functioning; however, his mother notes that she is beginning to worry about his school progress. He is not reading or spelling as well as his classmates, and he has been playing with the first- and second-grade children more than his peers. She provides school records and teacher comments that verify this concern. Which ONE of the following procedures would be most useful for ongoing tracking of this youngster’s progress:
  - A referral for a comprehensive neuropsychological evaluation that includes IQ, specific cognitive domains, academic achievement, and social-behavioral assessments
  - An IQ test to rule-out mental retardation
  - A meeting with the child’s teacher
  - No procedures for follow-up, but remember to ask about school and social functioning upon the next medical visit
2. The special education rate for children with CKD is no different from the national rate at this time. (True/False)
3. Based on current research, the prevalence of cerebral atrophy from imaging results in the general pediatric CKD population has been estimated to range between:
  - 10 – 25%
  - 30 – 45%
  - 50 – 65%
  - 70 – 85%
4. EEG abnormalities have been found to be prevalent in the pediatric CKD population at rates ranging between:
  - 0 – 25%
  - 25 – 50%
  - 50 – 75%
  - 75 – 100%
5. Which brain regions, structures, and pathways have been identified as vulnerable in children with CKD?
  - Periventricular white matter
  - Internal junctional border/watershed zones
  - Somatosensory cortex
  - All of the above
6. Adult survivors of childhood onset ESRD meet national expectations for educational achievement and employment. (True/False)
